# Multi-Objective Geometry Optimization of Additive-Manufactured Hexagonal Honeycomb Sandwich Beams Under Quasi-Static Three-Point Bending Loading

**DOI:** 10.3390/ma18040867

**Published:** 2025-02-17

**Authors:** Andres Cecchini, Marco Menegozzo, Emerson Roman

**Affiliations:** 1 Department of Mechanical Engineering, University of Memphis, Memphis TN 38152, USA; cecchini@memphis.edu; 2 Department of Mechanical Engineering, University of Puerto Rico at Mayaguez, Mayaguez, PR 00681, USA; emerson.roman@upr.edu

**Keywords:** sandwich beam, honeycomb, additive manufacturing, multi-objective optimization

## Abstract

This research paper presents the findings of a design optimization analysis conducted on additive-manufactured thermoplastic sandwich structures with hexagonal honeycombs subjected to quasi-static three-point bending. Based on experimental results, finite element analysis, and analytical models, the relationship between four selected design variables (i.e., cell wall length ratio, cell wall angle, cell wall thickness, and skin thickness) and the structure’s mass, flexural stiffness, and maximum load capacity was determined. The influence of each design variable on the aforementioned structural properties was mathematically represented using three scaling laws to formulate a multi-objective optimization problem. Two conflicting objective functions, one for the mass and the other for the reciprocal of the maximum load capacity, along with a nonlinear constraint equation for the minimum allowed flexural stiffness of the sandwich structure were developed. The optimal values of the design variables were determined using two optimization methods, the Pareto optimal front and genetic algorithm, and by applying the Improved Minimum Distance Selection Method (IMDSM). Optimized designs were obtained for different values of flexural stiffness. It was found that, independently of the stiffness constraint value, the optimal value of the cell wall length ratio was 0.2 and the optimal cell wall thickness was 1.4 mm, which correspond to the minimum cell wall length ratio and maximum cell wall thickness considered in this study, respectively. On the other hand, if higher flexural stiffness is required for the structure, both cell wall angle and skin thickness must be increased accordingly. Furthermore, an increase in flexural stiffness is accompanied by an increase in both the mass and maximum load capacity of the structure.

## 1. Introduction

Sandwich panels are efficient lightweight structures designed to mainly sustain out-of-plane loads, which have enormous potential applications in aerospace, marine, automobile, and wind turbine industries. Sandwich panels consist of two thin skins with high longitudinal stiffness and strength bonded to a thick lightweight core that separates the skins, increasing the flexural loading capability of the structure. Cellular or lattice geometries have been extensively used in the fabrication of sandwich structural components due to their superior stiffness and strength per unit weight. In addition to their load-carrying capability, sandwich structures can be designed and optimized to perform multiple simultaneous functions, increasing their overall efficiency and reducing their environmental impact. Lattice honeycomb cores can incorporate multifunctionalities such as piezoelectric actuators, self-healing, sensing, and energy storage. This is now possible due to new advancements in additive manufacturing (AM) technologies, including 3D printing and selective laser sintering (SLS), among others. Additive manufacturing also allows for the fabrication of multifunctional and multi-material honeycombs with complex topologies (e.g., auxetic re-entrant, truss, chiral, origami-inspired, bio-inspired, metamaterial, hierarchical, triply periodic minimal surface, etc.) in which the honeycomb cell parameters can be gradually varied in space (functionally graded), resulting in structural designs with optimized mechanical properties. With this technology, in the near future, sandwich panels will be produced faster at a relatively lower cost and with less post-fabrication processing than conventional manufacturing methods. Recent research has demonstrated that 3D printed thin-walled honeycombs fabricated with thermoplastic materials (e.g., polylactic acid, acrylonitrile butadiene styrene, polycarbonate, polyurethane, etc.) or metal alloys (e.g., aluminum, stainless steel, brass, etc.) exhibit a remarkable structural response to in-plane quasi-static compression [1], three-point bending [2,3,4,5], low-velocity impact [6], blast [7,8,9], and other loading conditions [10]. Numerous studies in the literature [11,12,13,14,15,16] present comparative analyses of different two- and three-dimensional lattice topologies or propose innovative designs [17,18,19,20], showing their corresponding advantages and disadvantages. The optimization of the topology or the geometric parameters in the design of 3D printed sandwich structures is currently a subject of increasing scientific research. Recently, single- and multi-objective optimization analyses of sandwich structures for a wide variety of core topologies and different structural performance parameters (e.g., stiffness, strength, mass, energy absorption, etc.) have been successfully conducted to identify the corresponding optimal values of the design variables [21,22,23,24,25]. The conventional hexagonal, truss, and auxetic (i.e., with a negative Poisson ratio) re-entrant honeycombs are among the most studied lattice structures. The auxetic re-entrant geometry has shown significant energy absorption but relatively low stiffness. The truss structure has relatively high stiffness, but its simple geometry does not leave room for optimization. Finally, the hexagonal honeycomb presents the benefits that its geometry is suitable for optimization (i.e., up to five of its dimensions can be selected as design variables) and relatively high stiffness values can be achieved. However, despite the benefits that AM has brought to the design of sandwich structures, current 3D printed parts made from polymers, metals, or composite materials contain intrinsic defects, which may cause anisotropic behavior, poor strength and damage tolerance, and premature failure [26,27,28,29]. Furthermore, the relationship between geometric parameters, manufacturing conditions, mechanical properties, and structural response is not yet fully understood. This gap in knowledge has limited the use of 3D printed components to non-critical structural applications. The implementation of 3D printed sandwich panels on high-performance structures will require innovative designs, materials with consistent and predictable properties, and efficient manufacturing processes such that the aforementioned limitations can be overcome.

The research presented in this paper aims to address one of the most fundamental challenges related to sandwich structures with 3D printed cellular honeycombs, i.e., to determine the relationship between the geometric parameters and the structural properties, particularly the mass, stiffness, and strength, in order to optimize the design. The analysis is exclusively focused on the special case of hexagonal honeycombs, even though the proposed approach is not limited to this particular geometry and may be applied to other lattice topologies. Once the relationships between the geometric parameters and the structural properties are known, the geometry can be optimized according to certain design requirements, in this case minimizing the mass of the structure while satisfying specific constraints in terms of stiffness and/or strength. To this end, a series of test specimens with different geometric values were manufactured from polylactic acid (PLA) bio-based thermoplastic material by fused deposition modeling (FDM) and tested under quasi-static three-point bending. The experimental results were used to develop three scaling laws in the forms of *m* = *f_m_* (*h/l*, *θ, t*, *t_f_*), *K* = *f_K_* (*h/l*, *θ, t*, *t_f_*), and *P_u_* = *f_P_* (*h/l*, *θ, t*, *t_f_*), where *m*, *K*, and *P_u_* represent the mass, the stiffness, and the failure load, respectively, and *h/l*, *θ, t*, and *t_f_* are the selected geometric design variables. These scaling laws were obtained using regression models based on the theory of cellular solids [30]. The geometry of the sandwich beams was optimized using Pareto front and genetic evolutionary multi-objective optimization algorithms in MATLAB^®^-R2020b. Additionally, computational finite element (FE) models were developed in the commercial software LS-DYNA^®^-R11.0 and experimentally validated: they were used to identify areas of high stress concentration and failure mechanisms.

## 2. Materials and Methods

### 2.1. Sandwich Beam Design Variables

**Figure 1:**
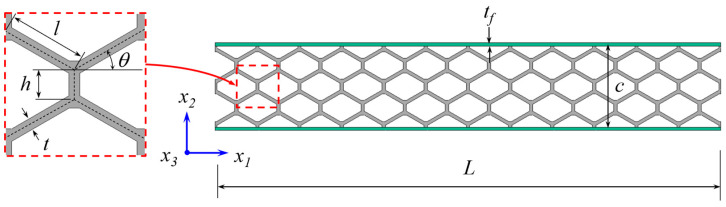
Honeycomb cell geometry and sandwich beam design variables.

In the initial phase of this research, four geometric parameters were selected as design variables for the sandwich beam specimens. These were the cell wall length ratio *h*/*l*, cell wall angle *θ*, cell wall thickness *t*, and skin thickness *t_f_* (see Figure 1). The selection of the geometry variation ranges was based on three constraining aspects: manufacturing, testing, and modeling. In particular, the cell wall ratio *h*/*l* and the cell angle *θ* were limited by the specimen’s length-to-height ratio, since these parameters control the thickness of the honeycomb. The combination of the highest values of *h*/*l* and *θ* produced a specimen’s length-to-height ratio of 0.25. Additionally, the cell wall thickness was limited by the quality of the manufacturing process and modeling considerations. The minimum cell wall thickness considered in this study was the smallest thickness printed with a 0.4 mm diameter nozzle that yielded acceptable dimensional tolerances. On the other hand, the maximum cell wall thickness was determined using the limiting value of the thin-walled honeycomb relative density, which was approximately 0.4 [30]. The minimum skin thickness followed the same consideration as for the cell wall thickness, and the maximum value was controlled with the maximum loading capacity of the testing system.

With the values of the design parameters specified, a MATLAB^®^ script was developed to automatically create and export the honeycomb geometry for manufacturing and modeling. This beam specimen represents a sandwich panel section with constant width, length, and span between supports. The results obtained from this analysis can be extended to a planar sandwich with any width, since the mass, stiffness, and maximum load capacity are linearly dependent on this dimension.

As a result, 16 sandwich beam designs were selected for manufacturing and testing. This was the number of specimens corresponding to two limiting values for each of the four design parameters (2^4^ = 16). The possibility of including a third intermediate value for each design variable was discarded since the number of specimens to be manufactured and tested would have been too large for the scope of this project (3^4^ = 81). Consequently, the 16 specimens listed in Table 1 were selected for manufacturing and testing. All the specimens had the same length *L* = 108 mm, same width *b* = 10 mm, and same number of cells along the *x*_1_-direction (*Nx*_1_ =12 cells) and *x*_2_-direction (*Nx*_2_ *=* 2 cells). The relative density and honeycomb thickness were calculated using Equations (1) and (2), respectively:

(1) $$\frac{\rho_{c}}{\rho_{s}}=\frac{t/l\left(h/l+2\right)}{2\cos\theta \left(h/l+\sin\theta \right)}$$

(2) $$c=2{Nx}_{2}l\left(h/l+\sin\theta \right)$$

where *ρ_c_* is the honeycomb density, and *ρ_s_* is the solid material density of the filament, [30]. The maximum relative density obtained was 0.383 for specimens # 3 and 4 (Table 1), which satisfied the constraint of the thin-walled honeycomb (less than 0.4) [30], whereas the core thickness varied between 16.6 mm and 26.9 mm.

### 2.2. Test Specimen Manufacturing

**Figure 2:**
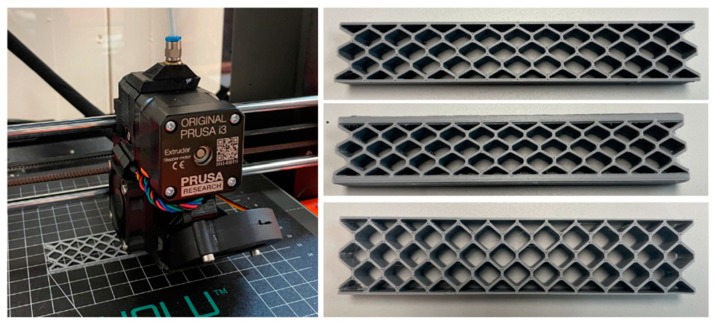
The 3D printing of sandwich beam test specimens.

The sandwich beam test specimens were manufactured by fused deposition modeling (FDM) using an Original Prusa i3 MK3S + 3D-Printer. Figure 2 shows the printing orientation and three sandwich specimens. As expected, the printing quality of the samples was shown to be highly dependent on the printing temperature, speed, and nozzle material. The moisture content of the printing material turned out to be another critical factor on the quality of the parts. All manufacturing parameters (i.e., nozzle diameter, temperature, speed, printing orientation, etc.) were kept constants for all the specimens to maintain consistency. The printing material for the sandwich specimens was polylactic acid (PLA), which is a bioplastic-based 3D printing material filament that provides acceptable printing quality, mechanical properties, manufacturing speed, and reliability. The specimens were manufactured using a layer thickness of 0.1515 mm, and the number of layers was 66. The nozzle temperature was 190 °C, and the wall speed was set to 10 mm/s, whereas the infill speed was 50 mm/s.

### 2.3. Material Characterization

**Figure 3:**
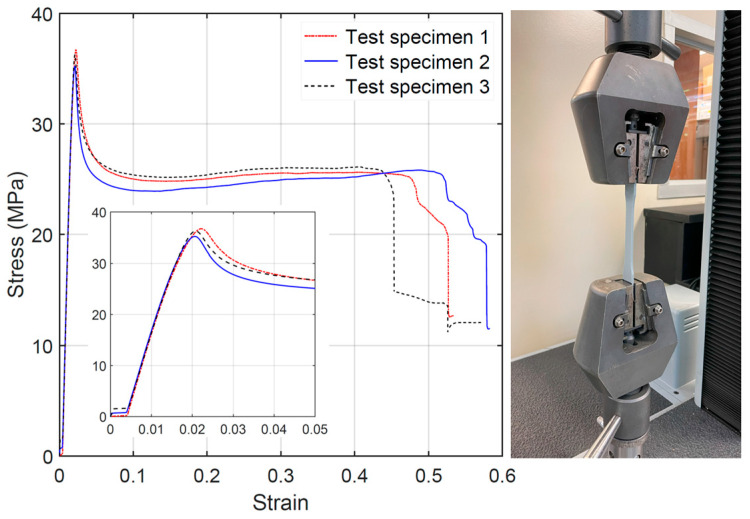
Tension test of PLA dogbone specimens: stress–strain curve (with the elastic branch zoomed-in on) and experimental setup.

The mechanical behavior of the PLA printing material was characterized through the tension testing of dogbone specimens according to ASTM standard D638 [31]. The dogbone specimens were printed with the filaments aligned with the direction of the applied stress (i.e., the longitudinal direction). The dogbone dimensions were selected based on [31]: 115 mm gauge long, 13 mm wide, and 3 mm thick. As shown in Figure 3, the mechanical response of the three specimens was very consistent up to the fracture point: a linear-elastic regime with a well-defined yielding point followed by a sudden drop in stress and a quite uniform stress plateau. The average properties of the PLA material are listed in Table 2.

### 2.4. Three-Point Bending Test

**Figure 4:**
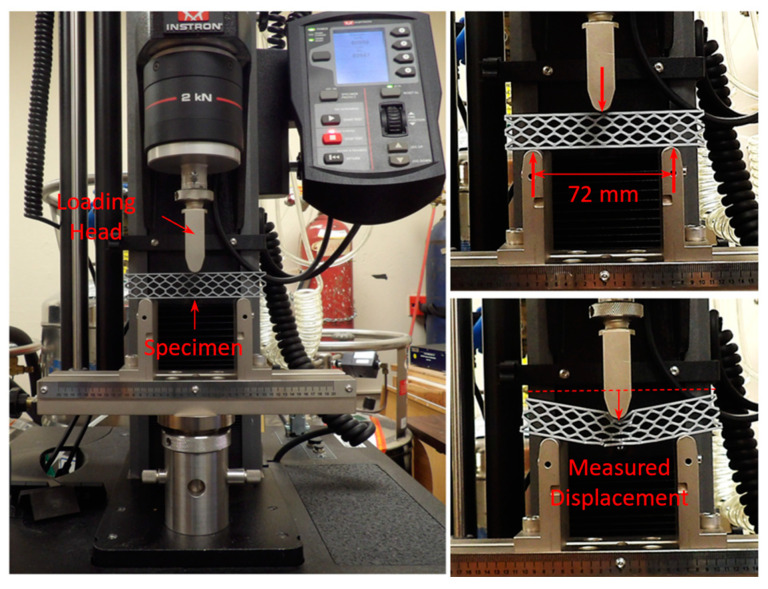
Quasi-static three-point bending test of 3D printed specimen.

Quasi-static three-point bending tests of the 3D printed sandwich specimens were carried out using an Instron^®^ universal testing system (Norwood, MA, USA) with a maximum load capacity of 2 kN (see Figure 4). The Instron testing system is appropriate for testing thermoplastic materials. Three test specimens of each geometry were tested under quasi-static three-point bending. The tests were conducted at a constant loading rate of 0.008 mm/s with a maximum displacement of 20 mm, in accordance with standard ASTM C393 [32]. During the tests, the specimen’s deformation mechanism was recorded, and the force versus displacement curves were obtained. These results were used later to determine the relationship between the mechanical properties of the specimens (e.g., stiffness, mass, and maximum initial force) and the four design geometric parameters (*h*/*l*, *θ*, *t*, *t_f_*). The results proved to be highly consistent and repeatable.

### 2.5. Finite Element Modeling

**Figure 5:**
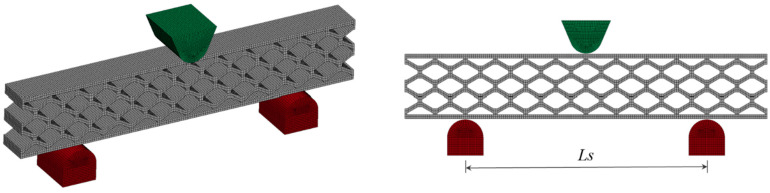
FE model of quasi-static three-point bending (implicit) analysis.

Finite element (FE) models of the quasi-static three-point-bending test were developed using the commercial software LS-DYNA^®^, as shown in Figure 5. Implicit analysis (time-independent) was conducted and validated against experimental data. Both honeycomb and skins were modeled using eight-node linear solid elements with an approximate size of 0.4 mm. The interface between the honeycomb and each skin was assumed to be perfectly bonded (i.e., no delamination was allowed). Friction contacts were defined between the loading head and the upper skin and between the lower skin and the fixed supports. The material behavior of the sandwich specimens was modeled as elastic–perfectly plastic with the mechanical properties listed in Table 2. The material of the loading head and the fixed support was assumed to be rigid. The loading head’s maximum vertical displacement was set to 5 mm, which was sufficient for capturing the initial region of the structural response (linear elastic regime and maximum initial reaction force). The span of the beam between supports was *L_s_* = 72 mm. The results demonstrated very good correlation with experiments.

## 3. Results and Discussion

### 3.1. Three-Point Bending Test Results

**Figure 6:**
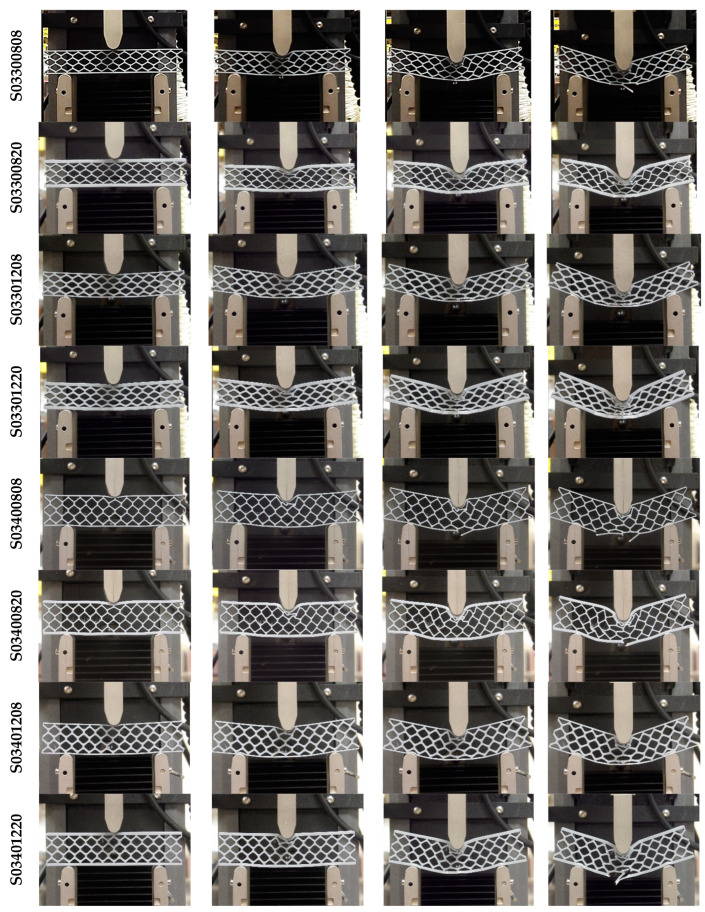
Quasi-static three-point bending test of 3D printed specimens (h/l = 0.3).

**Figure 7:**
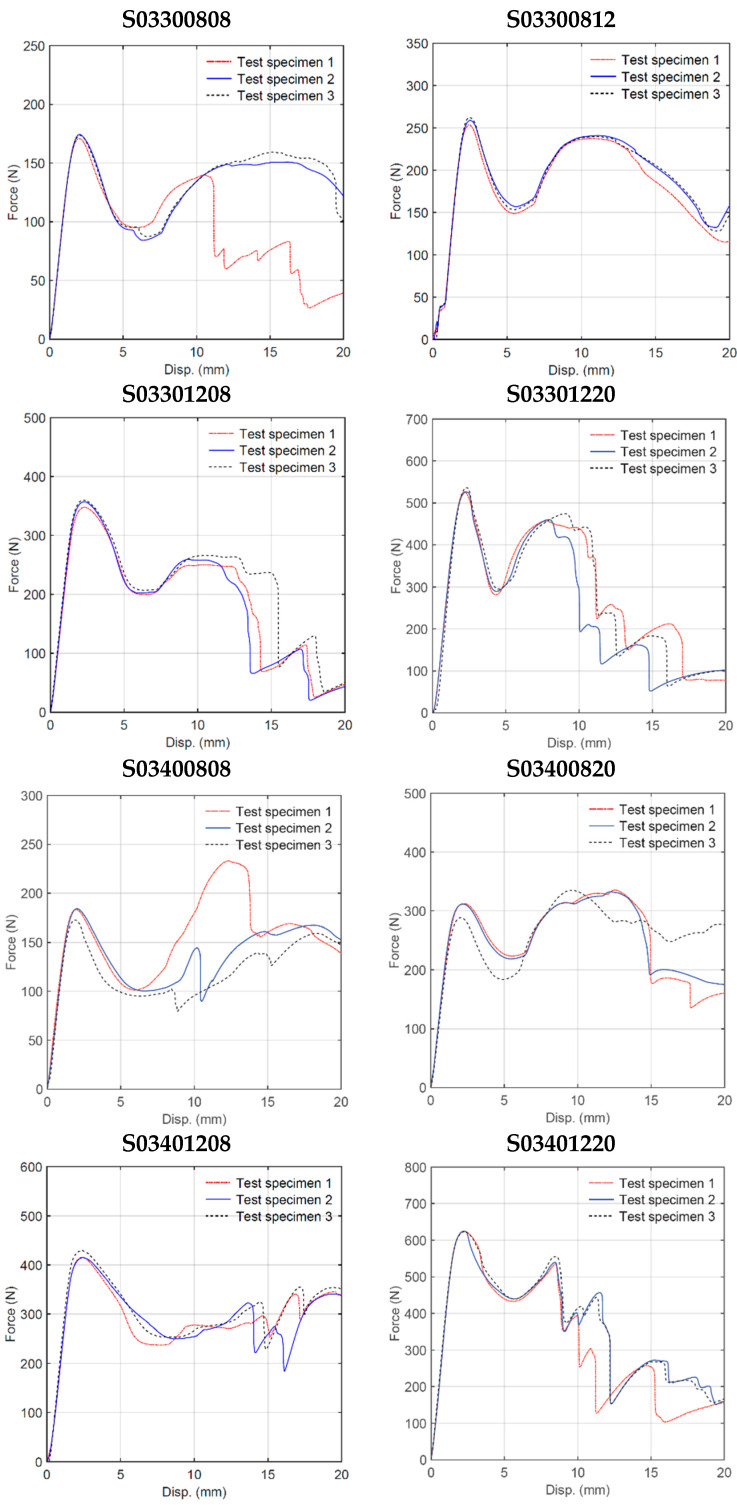
Quasi-static three-point bending test force–displacement curves (h/l = 0.3).

**Figure 8:**
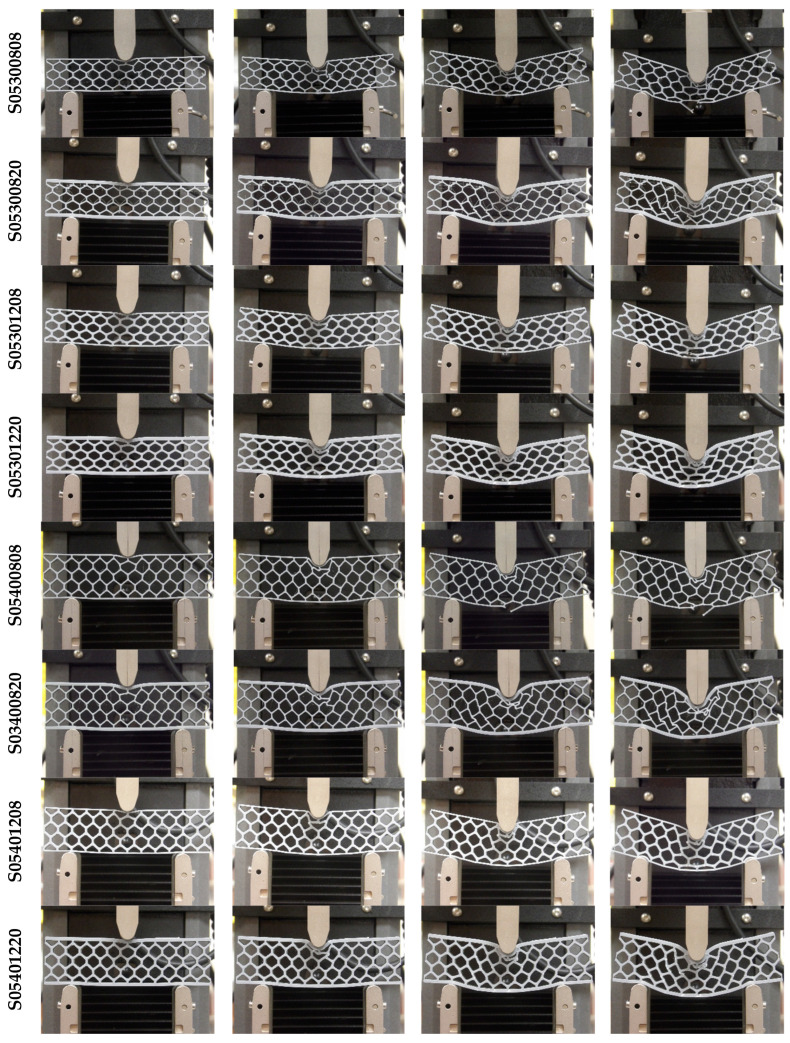
Quasi-static three-point bending test of 3D printed specimens (h/l = 0.5).

**Figure 9:**
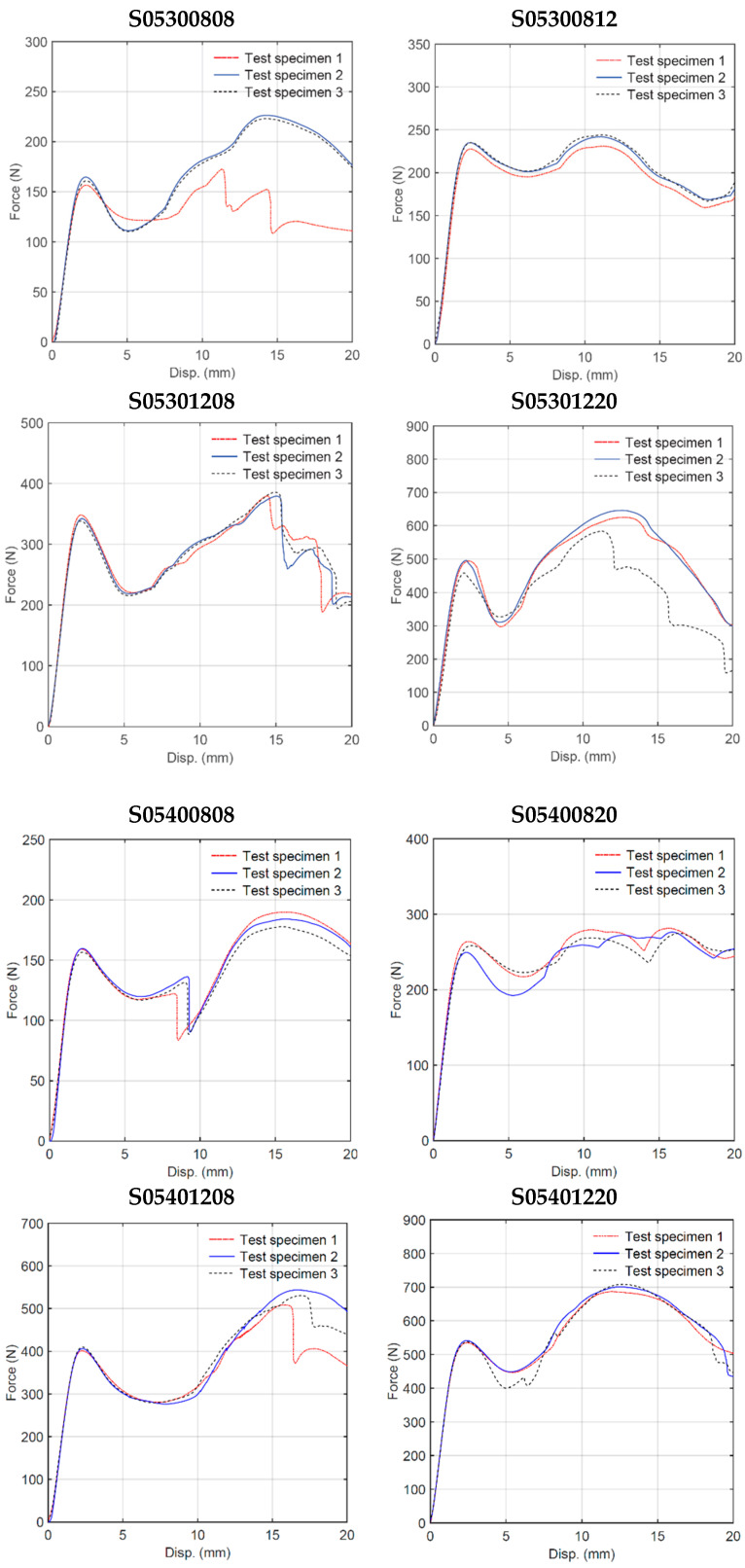
Quasi-static three-point bending test force–displacement curves (h/l = 0.5).

Quasi-static three-point bending tests were conducted on each of the 16 sandwich beam specimens described in Table 1. Three test specimens of each geometry were manufactured by FDM and tested following a random selection process. Figure 6 shows the deformation behavior of the specimens with cell wall length ratio *h/l* = 0.3. For some specimens, the only failure mode observed was plastic deformation, whereas for other specimens, plastic deformation was followed by honeycomb and/or skin fracture. For specimens with a particular geometry, the results were very consistent up to the first 5 mm displacement, as shown in Figure 7. After this initial displacement, some specimens (e.g., S03300808, S03400808, and S03400820) experienced failure by fracture at different locations, which is evidenced by the sudden drop in the force versus displacement curve. Figure 8 and Figure 9 show the deformation behavior and force versus displacement curves for *h/l* = 0.5, respectively. In this case, also, the initial failure mode for all specimens was the plastic deformation of the honeycomb, and in some cases (e.g., S05300808 and S05400808), skin fracture was observed. As shown in Figure 9, the results of specimens with a particular geometry were observed to be very consistent as well.

**Figure 10:**
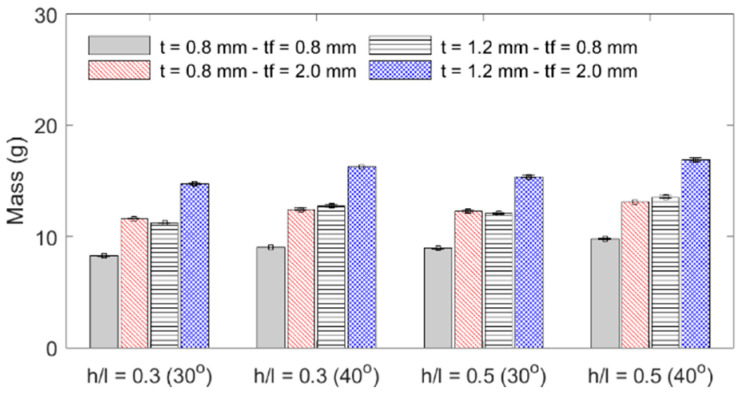
Specimen mass.

**Figure 11:**
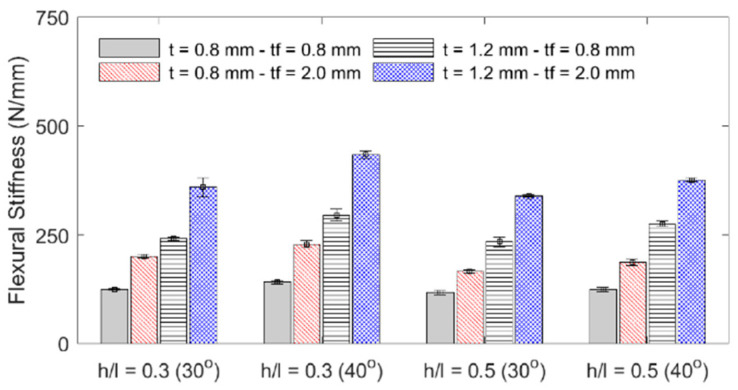
Specimen stiffness.

**Figure 12:**
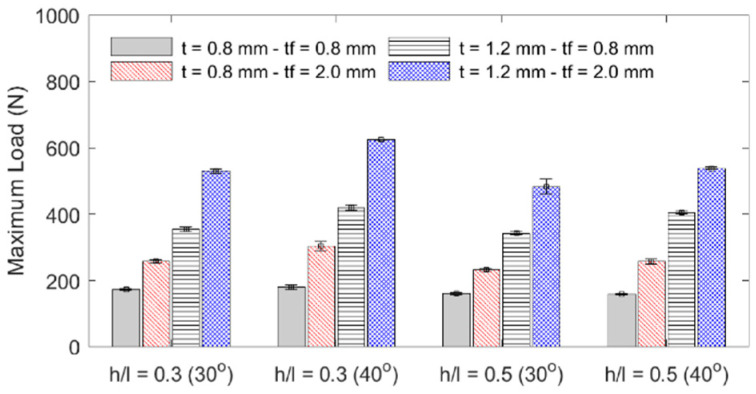
Specimens’ maximum initial load.

**Figure 13:**
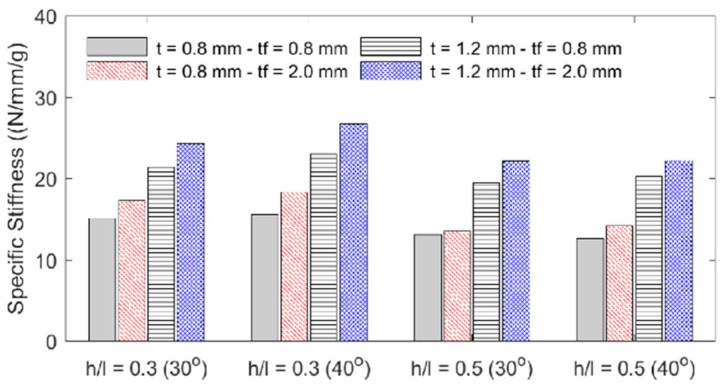
Specimens’ specific stiffness.

The influence of each geometric parameter (*h*/*l*, *θ*, *t*, *t_f_*) on the sandwich beam’s mass, flexural stiffness, maximum initial reaction force, specific flexural stiffness (stiffness per unit mass), and deformation energy up to the 5 mm displacement is summarized in Table 3 and graphically described in the bar plots shown in Figure 10, Figure 11, Figure 12 and Figure 13. The bar plots represent the average value, whereas the error bars represent the corresponding standard deviation in the experimental data. From the interpretation of these results, the following remarks can be drawn: (1) The mass of each specimen is highly dependent on both the honeycomb cell wall and skin thickness (*t* and *t_f_*), as expected, but it is not significantly influenced by either the cell wall length ratio (*h*/*l*) or the cell wall angle (*θ*); however, it can be noted that the general behavior is that an increase in value of any of the four design variables results in an increase in mass. (2) The flexural stiffness increases with an increasing cell wall angle (*θ*), honeycomb thickness (*t*), and skin thickness (*t_f_*) but decreases with an increasing cell wall length ratio (*h*/*l*), which is consistent with the fact that the theoretical elastic modulus of the core along the *x*_1_-direction is inversely proportional to *h*/*l* [30]. (3) The maximum initial reaction force behaves in the same manner as the flexural stiffness, and (4) the specific stiffness increases with both the honeycomb cell wall and skin thickness and slightly decreases with the cell wall length ratio, and it is not significantly affected by changes in the cell wall angle.

### 3.2. Finite Element Modeling Results

**Figure 14:**
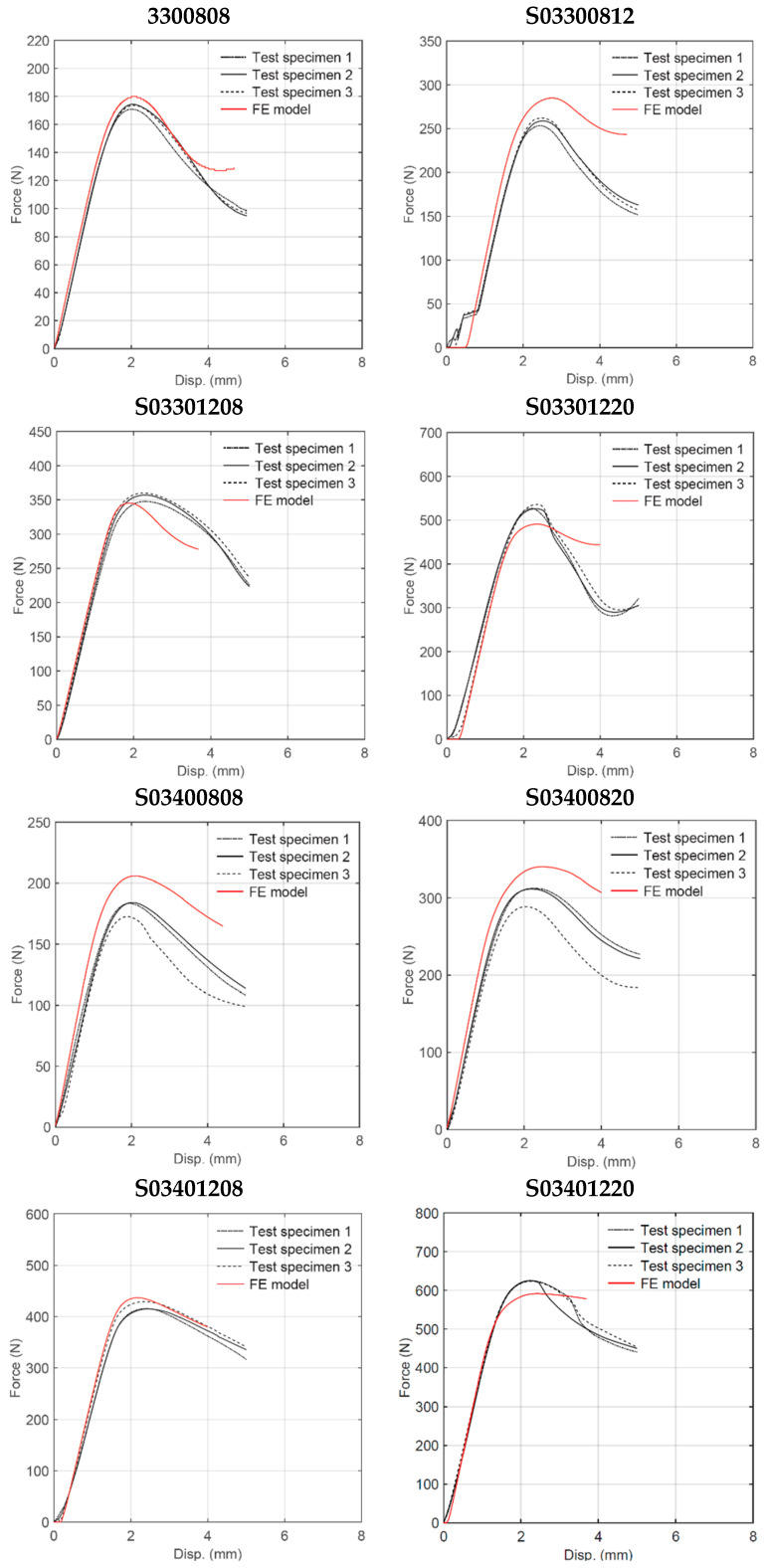
Experimental and numerical force–displacement curves (h/l = 0.3).

**Figure 15:**
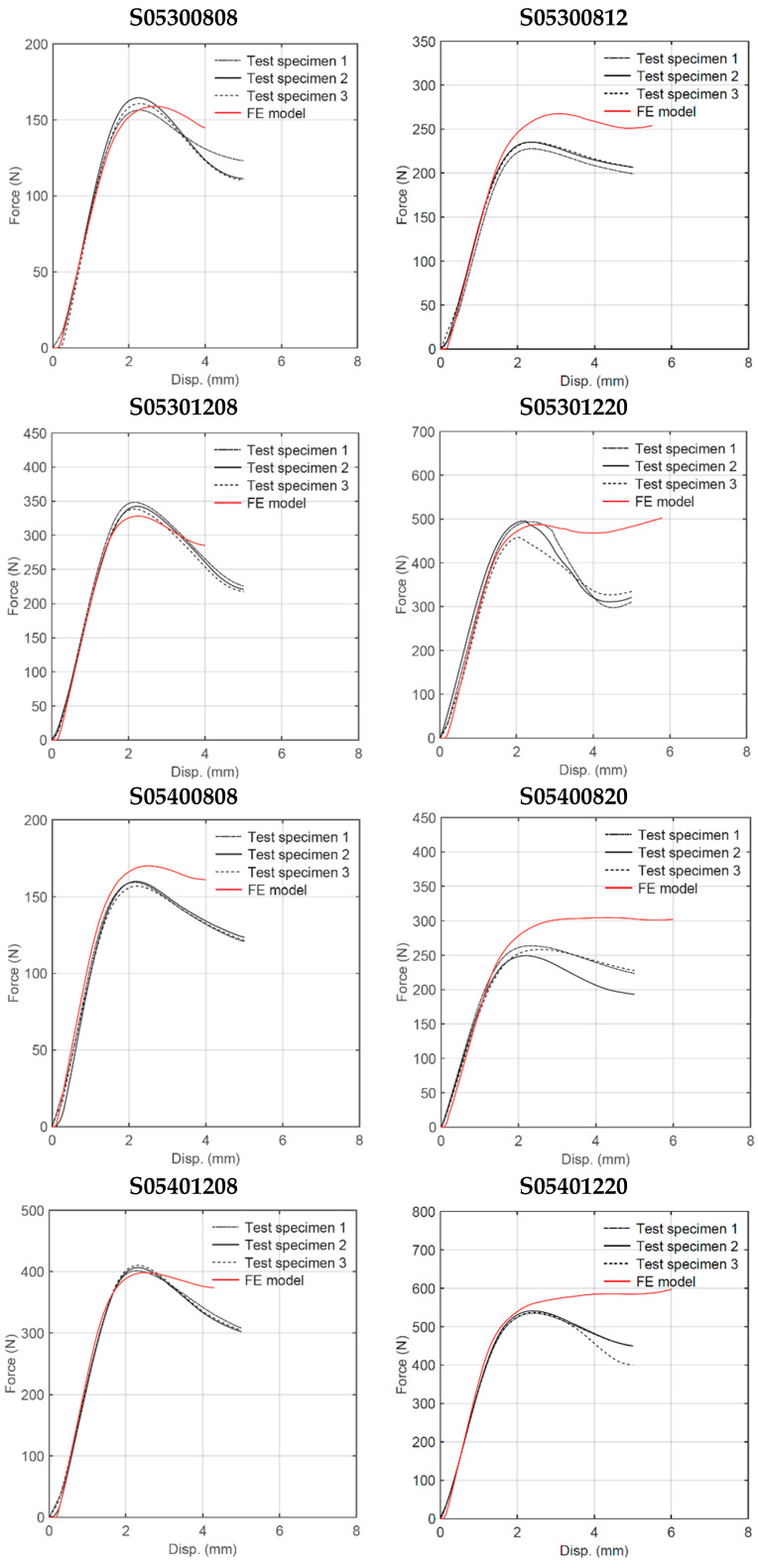
Experimental and numerical force–displacement curves (h/l = 0.5).

**Figure 16:**
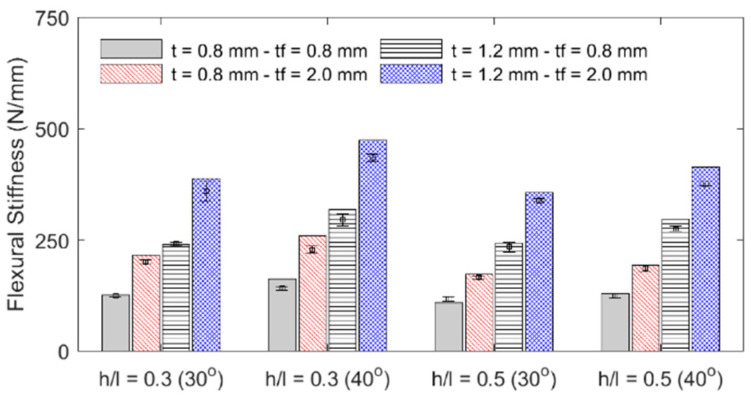
FE (bars) vs. experimental (error bars) stiffness.

**Figure 17:**
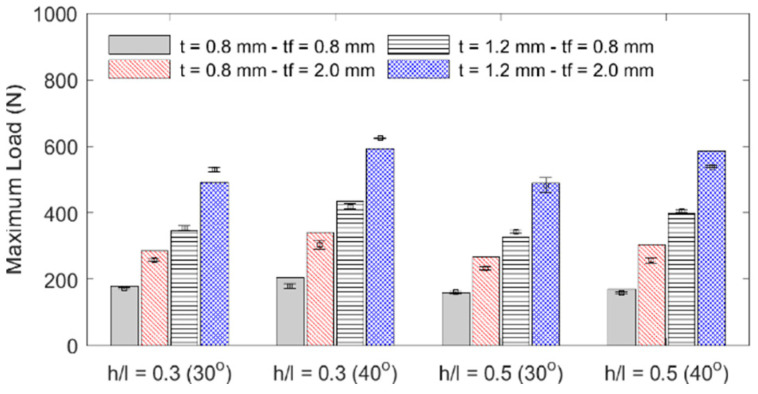
FE (bars) vs. experimental (error bars) maximum initial load.

Nonlinear implicit finite element (FE) analysis was conducted and experimentally validated for each sandwich beam geometry. The main goal of the FE analysis was to develop a modeling technique able to predict the structural response of sandwich beam specimens with geometric parameters different from those manufactured and tested. The maximum vertical displacement was set to 5 mm. The comparison between experimental and numerical results can be seen in Figure 14 and Figure 15 for *h*/*l* = 0.3 and *h/l* = 0.5, respectively. The curves were shifted to synchronize the displacements of the FE and experimental specimens. Table 4 summarizes FE predictions for the maximum load, stiffness, deformation energy up to a 5 mm displacement, and mass. The results are compared with average experimental measurements, and the error in percentage is reported within parentheses. The overall trend in the model behavior is that the predicted flexural stiffness shows very good correlation with the value measured experimentally (see Figure 16). The maximum load, on the other hand, was well predicted in most cases but it was over-predicted in other cases (see Figure 17). This may be attributed to the fact that the elapsed time between manufacturing and testing was not maintained constant and those specimens that were exposed for more time to environmental conditions such as ambient moisture may have experienced a decrease in yield strength.

### 3.3. Parametric Analysis

To quantitatively determine the relationship between the sandwich beam’s geometric parameters and their structural mass, flexural stiffness, and maximum initial load, a parametric analysis was performed using the experimental data. In addition to the original 16 test specimens defined in Table 1, 8 additional geometries were manufactured and tested, 6 central (i.e., withing the original design variable interval) and 2 axial data points (i.e., outside the original design variable interval). This was required to include nonlinear effects on the regression models. The central and axial data points shown in Table 5 were determined based on design and analysis of experiments theory [33].

**Figure 18:**
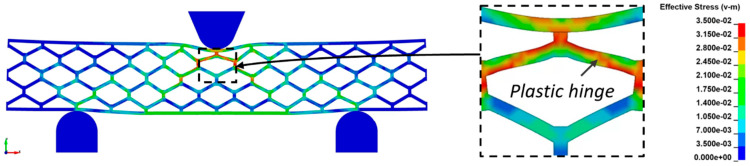
Formation of plastic hinge in inclined honeycomb cell wall below the loading head.

The regression models of Equations (3)–(5) were developed using analytical expressions for the mass, stiffness, and maximum load, respectively, according to Gibson and Ashby [30]:

(3) $$m=2\rho_{s}bLt_{f}+\rho_{c}bLc$$

(4) $$K=\frac{B_{1}{\left(EI\right)}_{eq}B_{2}{\left(AG\right)}_{eq}}{B_{1}L_{s}{\left(EI\right)}_{eq}+B_{2}{L_{s}}^{3}{\left(AG\right)}_{eq}}$$

(5) $$P_{pl}=\frac{\sigma_{ys}bt^{2}}{2l\sqrt{\left(1-\sin^{2}\theta \right)}}$$

where *ρ_c_* represents the density of the honeycomb given by Equation (2), *B*_1_ and *B*_2_ are constants associated with the beam supports and loading condition, and (*EI*)_eq_ and (*AG*)_eq_ are the equivalent flexural stiffness and equivalent transverse shear stiffness, respectively. Equation (5) is derived from the fact that the maximum initial load is associated with the plastic collapse of the cell walls located in the vicinity of the loading head. Therefore, *P*_pl_ is the magnitude of the applied load required to cause the formation of plastic hinges in the inclined walls of the cell located below the loading head, as shown in Figure 18 obtained from FE analysis. *σ_ys_* represents the yield strength of the cell wall materials.

Furthermore, (*EI*)_eq_ and (*AG*)_eq_ are given by Equations (6) and (7), respectively, where *E_s_* represents the elastic modulus of the skin material, *E_c_* is the elastic modulus of the honeycomb along the *x*_1_-direction, and *G_c_* is the shear modulus of the honeycomb on the *x*_1_-*x*_2_ plane. According to Gibson and Ashby [30], *E_c_* and *G_c_* can be expressed as in Equations (8) and (9), respectively.

(6) $${\left(EI\right)}_{eq}=E_{s}bt_{f}^{3}/6+2E_{s}bt_{f}{\left(c/2\right)}^{2}+E_{c}bc^{3}/12$$

(7) $${\left(AG\right)}_{eq}=bcG_{c}$$

(8) $$E_{c}=\frac{E_{s}\cos\theta t^{3}}{l^{3}\left(h/l+\sin\theta \right)\sin^{2}\theta }$$

(9) $$G_{c}=\frac{E_{s}\left(h/l+\sin\theta \right)t^{3}}{l^{3}{\left(h/l\right)}^{2}\left(1+2h/l\right)\cos\theta }$$

After substituting Equations (1) and (2) into Equation (3) and rearranging terms, the regression model for the specimen’s mass was obtained and is given by Equation (10):

(10) $$m=A_{1}t_{f}+\frac{A_{2}t\left(h/l\right)+A_{3}t}{\sqrt{\left(1-\sin^{2}\theta \right)}}$$

where *A*_1_, *A*_2_, and *A*_3_, are coefficients to be determined using the three-point bending experimental data. Similarly, in combining Equation (4) with Equations (6)–(9), the regression model described in Equation (11) was developed for flexural stiffness:

(11) $$c=C_{1}\left(h/l\right)+C_{2}\sin\theta \;E_{c}=\frac{C_{4}\sqrt{\left(1-\sin^{2}\theta \right)}t^{3}}{\left(h/l+\sin\theta \right)\sin^{2}\theta }\;G_{c}=\frac{C_{3}ct^{3}}{{\left(h/l\right)}^{2}\left(1+h/l\right)\sqrt{\left(1-\sin^{2}\theta \right)}}\;{\left(EI\right)}_{eq}=t_{f}^{3}+t_{f}c^{2}+E_{c}c^{3}\;{\left(AG\right)}_{eq}=cG_{c}\;K=\frac{C_{5}{\left(EI\right)}_{eq}{\left(AG\right)}_{eq}}{{\left(EI\right)}_{eq}+{\left(AG\right)}_{eq}}$$

where *C_i_*, *i* = 1, …, 5, are coefficients to be determined experimentally as well. Furthermore, based on Equation (5), the maximum initial load *P*_pl_ seems to be independent of both the cell wall length ratio *h*/*l* and the skin stiffness *t_f_*. This is because Equation (5) was developed for honeycombs under compression only and not for honeycomb sandwich beams under three-point bending. Therefore, Equation (5) was modified accordingly to account for the difference in the loading condition and to be consistent with the experimental results. Two additional terms were included, one associated with *h*/*l* and the other associated with *t_f_*. Thus, the regression model for the maximum initial force given in Equation (12) was obtained.

(12) $$P_{max}=D_{1}{\left(h/l\right)}^{D_{4}}+\frac{D_{2}t^{2}}{\sqrt{\left(1-\sin^{2}\theta \right)}}+D_{3}{t_{f}}^{D_{5}}$$

where *D_i_*, *i* = 1, …, 5, also are experimental coefficients corresponding to this regression model. The results of the regression analysis are listed in Table 6. This includes the regression coefficients, *R*-squared (*R*^2^), and root mean squared error (*RMS*) corresponding to each regression model.

#### 3.3.1. Effect of Cell Wall Ratio

**Figure 19:**
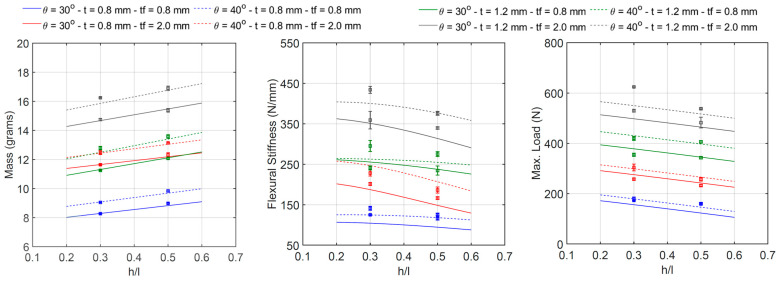
Relationship between h/l and beam mass, flexural stiffness, and maximum initial load.

The relationship between the cell wall length ratio *h*/*l* and the specimen’s mass, flexural stiffness, and maximum initial load is shown in Figure 19. Each line corresponds to the intersection between the four-dimensional regression surface and a plane with the constant values of the angle *θ*, thickness *t*, and thickness *t_f_*. Experimental data are also plotted as error bars. The regression models confirm the remarks stated in Section 3.1: an increase in the ratio h/l (i.e., from 0.3 to 0.5) yields a slight increase in mass (between 4.0 and 8.7 percent) and a reduction in both stiffness (between 2.7 and 18.4 percent) and maximum load (between 3.3 and 15.4 percent). The relationship between *h*/*l* and the mass and the maximum load is practically linear, whereas the relationship between *h*/*l* and the stiffness presents some degree of nonlinearity.

#### 3.3.2. Effect of Cell Wall Angle

**Figure 20:**
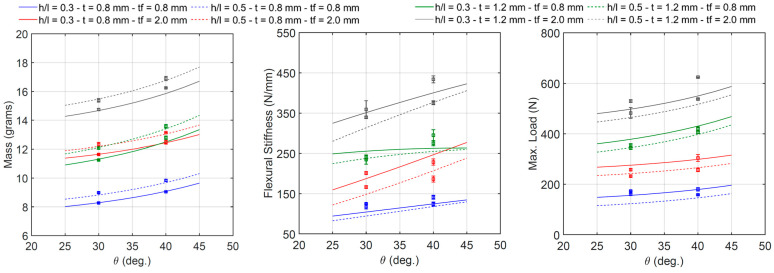
Relationship between θ and beam mass, flexural stiffness, and maximum initial load.

The effect of the cell wall angle *θ* and the specimen’s mass, flexural stiffness, and maximum initial load is shown in Figure 20. Each line corresponds to the intersection between the four-dimensional regression surface and a plane with the constant values of the cell wall length ratio *h/l*, thickness *t*, and thickness *t_f_*. The regression results also confirm what was previously mentioned in Section 3.1: the specimen’s mass increases between 6.8 and 13.6 percent, stiffness increases between 5.5 and 22.4 percent, and maximum load increases between 4.1 and 18.4 percent, with an increasing angle *θ* from 30°to 40°. The relationship between these parameters is clearly nonlinear.

#### 3.3.3. Effect of Cell Wall Thickness

**Figure 21:**
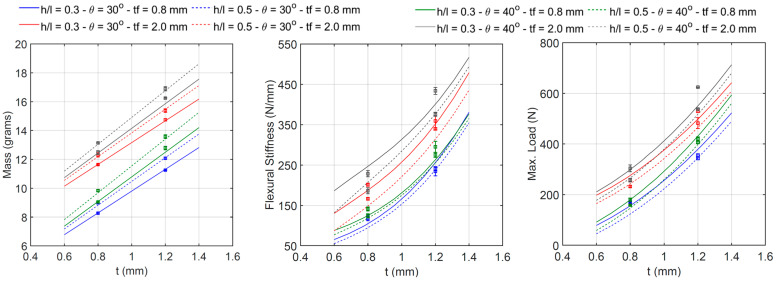
Relationship between t and beam mass, flexural stiffness, and maximum initial load.

Figure 21 shows that the mass, the stiffness, and the maximum load are all highly dependent on the cell wall thickness *t*. An increase in this parameter (from 0.8 to 1.2 mm) yields a significant increment in the three properties of the specimens, between 24.9 and 41.3 percent in mass, between 78.6 and 121.9 percent in stiffness, and between 105.2 and 156.1 percent in the maximum load. The relationship between *t* and the mass is practically linear, whereas some degree of nonlinearity can be observed for the relationship between *t* and both stiffness and maximum load. Each line corresponds to the intersection between the four-dimensional regression surface and a plane with the constant values of the *h*/*l* ratio, angle *θ*, and thickness *t_f_*.

#### 3.3.4. Effect of Skin Thickness

**Figure 22:**
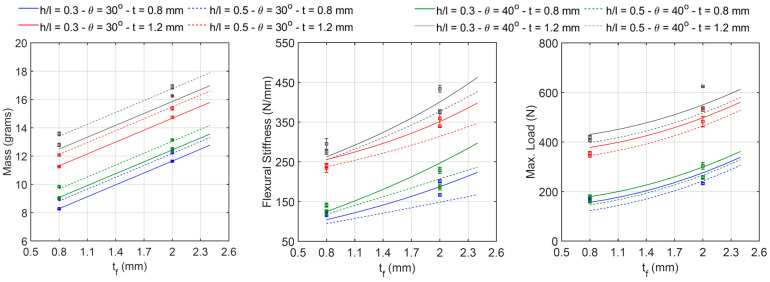
Relationship between t_f_ and beam mass, flexural stiffness, and maximum initial load.

The effect of the skin thickness *t_f_* on the specimen’s mass, flexural stiffness, and maximum initial load is similar to that between the cell wall thickness *t* and the specimen’s mechanical properties (see Figure 22). There is a relatively high dependency between them, and an increase in thickness *t* results in a significant improvement in all the specimen’s properties, particularly between 24.4 and 40.6 percent in mass, between 36.6 and 62 percent in stiffness, and between 32.4 and 69 percent in the maximum load. The relationship between *t_f_* and the mass is almost linear, whereas some degree of nonlinearity can be observed regarding the relationship between *t_f_* and both the stiffness and maximum load. Each line corresponds to the intersection between the four-dimensional regression surface and a plane with the constant values of the *h*/*l* ratio, angle *θ*, and thickness *t*.

### 3.4. Geometry Optimization

The parametric analysis described in the previous section provided the understanding of the relationship between the selected design variables (*h*/*l*, *θ*, *t*, *t_f_*) and the sandwich beam’s mechanical properties (i.e., mass, flexural stiffness, and maximum load capacity). In combining Equations (10)–(12), the multi-objective optimization (MOO) problem described by Equation (13) was defined.

(13) $$\left\{\begin{array}{c} \begin{array}{c} min.\;\;m=A_{1}t_{f}+\frac{A_{2}th/l+A_{3}t}{{\left[1-\sin^{2}\theta \right]}^{1/2}}\\ {P_{max}}^{-1}={\left[D_{1}{\left(h/l\right)}^{D_{4}}+\frac{D_{2}t^{2}}{{\left(1-\sin^{2}\theta \right)}^{1/2}}+D_{3}{t_{f}}^{D_{5}}\right]}^{-1} \end{array}\\ \begin{array}{c} \begin{array}{c} s.t.\;K=\frac{C_{5}{\left(EI\right)}_{eq}{\left(AG\right)}_{eq}}{{\left(EI\right)}_{eq}+{\left(AG\right)}_{eq}}\geq K_{min} \end{array}\\ \begin{array}{c} 0.2\leq h/l\leq 0.6\\ 25{}^{\circ}\leq \theta \leq 45{}^{\circ}\\ 0.6\;mm\leq t\leq 1.4\;mm\\ 0.8\;mm\leq t_{f}\leq 2.4\;mm \end{array} \end{array} \end{array}\right.$$

**Figure 23:**
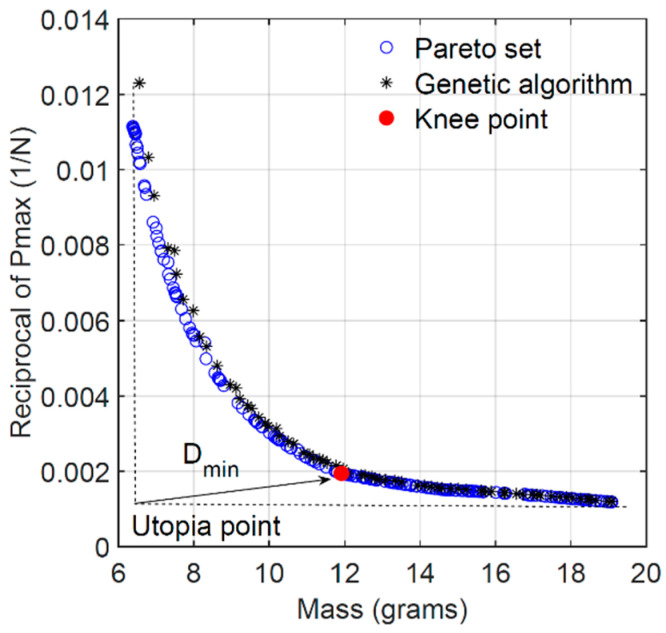
Pareto optimal front and GA (*K*_min_ = 0).

**Figure 24:**
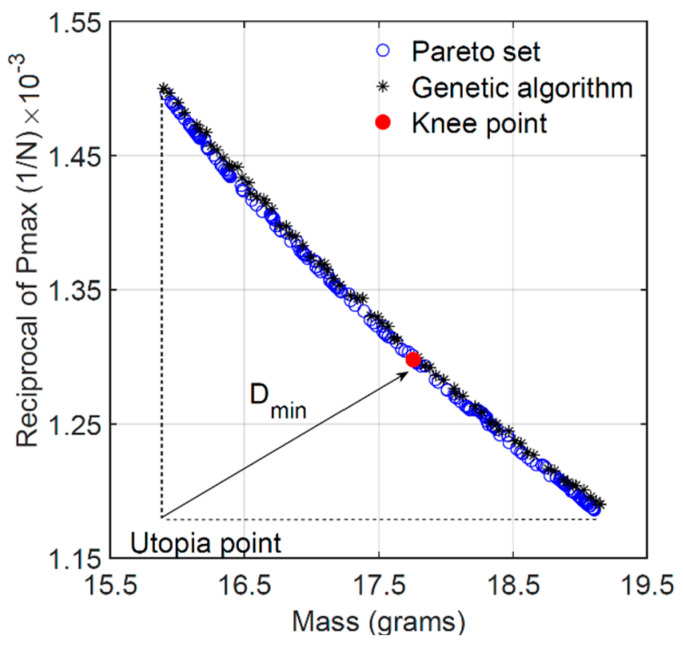
Pareto front and GA (*K*_min_ = 500 N/mm).

The goal is to find the optimal values of the design variables that simultaneously minimize the mass of the sandwich beam and maximize its load carrying capacity (i.e., minimize the reciprocal of the maximum load). The two conflicting goals are defined as objective functions. Additionally, the solution of the MOO problem is subjected to the following restrictions or constraints: the flexural stiffness of the beam must satisfy a certain minimum value *K*_min_, and each design variable must remain within its corresponding interval. For the solution of the MOO problem of Equation (13), two optimization algorithms were investigated and compared using MATLAB^®^: the Pareto optimal front and genetic algorithm. The Pareto optimal front consists of a set of optimal solutions in which every solution in the set meets the requirements of the objective functions and satisfies the problem constraints. However, determining which solution in the set is the most satisfactory (the “knee point”) still requires further selection [24]. Genetic algorithms solve optimization problems by randomly searching by mutation and crossover among population members. In this work, the genetic algorithm used in MATLAB^®^ also provided a Pareto front of multiple fitness solutions using a genetic algorithm. The evaluation of the Pareto solutions and the determination of the knee point was carried out using a normalized-based method known as the Improved Minimum Distance Selection Method (IMDSM), given by Equation (14):

(14) $$D_{min}=min\left(\sqrt{\sum_{i=1}^{n}{\left[\frac{f_{ci}(x)}{min\left(f_{i}(x\right))}-1\right]}^{2}}\right)$$

where *n* is the number of objective functions, *f_ci_*(***x***) is the *i*th objective value in the *c*th Pareto solution, and *D*_min_ is the minimum or the shortest distance from the “utopia point” to the knee point. The solution corresponding to the minimum distance *D*_min_ in the Pareto set was taken as the optimal solution of the MOO problem since it was considered to be best tradeoff between flexural stiffness and maximum load capacity. Figure 23 shows the Pareto front and genetic algorithm solutions sets consisting of 200 points each for the special case of the no-stiffness constraint (i.e., *K*_min_ = 0). The most satisfactory solution is also identified as the “knee point”. Both algorithms seem to generate a similar set of solutions except for their distribution on the solution domain. The convex shape of the solutions set is characteristic of the two conflicting objective functions. If the mass of the beams decreases, the maximum load capacity also decreases. Hence, the knee point was selected as the most satisfactory solution within the set that could be obtained for this case scenario. The corresponding optimal values of the design variables are listed in Table 7. The optimal sandwich beam geometry with no stiffness constraint is given by the minimum acceptable ratio h/l = 0.2, angle *θ* = 25.1°, skin thickness *t_f_* = 0.8 mm, and maximum acceptable cell wall thickness *t* = 1.4 mm. The corresponding properties are mass *m* = 11.91 g, load capacity *P*_max_ = 514.8 N, and flexural stiffness *K* = 390.3 N/mm. On the other hand, the Pareto front and genetic algorithm solutions sets with the stiffness nonlinear constraint of *K*_min_ = 500 N/mm is shown in Figure 24. In this case, the optimal solution for the beam geometry is given by the minimum h/l = 0.2, the maximum cell wall and skin thickness t = 1.4 mm and *t_f_* = 2.4 mm, and intermediate angle *θ* = 37.4°. Between these two opposing case scenarios (i.e., 0 ≤ *K*_min_ ≤ 500 N/mm), the optimal cell wall length ratio remains constant at its minimum value of 0.2, and the optimal cell wall thickness also remains constant at its maximum allowable value of 1.4 mm. Increasing the value of the stiffness constraint causes the optimal cell wall angle to increase from its minimum allowable value to a maximum for *K*_min_ = 400 N/mm and then to decrease again to 37.4° for *K*_min_ = 500 N/mm. The optimal skin thickness increases from its minimum to its maximum allowable value when increasing the value of the minimum allowed stiffness in the constraint equation. Furthermore, an increase in the minimum allowed stiffness in the constraint equation is accompanied by an increase in both mass and maximum load capacity *P*_max_ of the structure.

## 4. Conclusions

The research presented in this paper addresses one of the most fundamental challenges related to the design and optimization of sandwich structures with 3D printed hexagonal honeycombs, which is to determine the relationship between the geometric design parameters and the structure’s mass, flexural stiffness, and maximum load capacity. Through experimental quasi-static three-point bending tests, finite element analysis, and analytical models, three scaling laws in the forms of *m* = *f_m_* (*h/l*, *θ, t*, *t_f_*), *K* = *f_K_* (*h/l*, *θ, t*, *t_f_*), and *P_u_* = *f_P_* (*h/l*, *θ, t*, *t_f_*) were developed. From this analysis, the following were found: (1) the mass of the structure increases with increasing the value of any of the four design variables, particularly cell wall thickness (41.3 percent increment) and skin thickness (40.6 percent increment); (2) both the flexural stiffness and maximum load capacity also increase with increasing values of the design variables (up to 121 and 156 percent, respectively) except for the honeycomb cell wall length ratio (up to 18.4 and 15.4 percent reduction, respectively). This indicates that the optimization of a sandwich structure with this particular honeycomb geometry will require the value of *h*/*l* to be reduced as much as possible. To obtain the optimal values of the design variables, a multi-objective optimization (MOO) problem was defined based on two conflicting objective functions, one for the mass and the other for the reciprocal of the maximum load capacity. Additionally, a nonlinear constraint equation was developed for the minimum allowed flexural stiffness of the sandwich structure. The aforementioned MOO problem was solved using two different optimization methods, the Pareto optimal front and genetic algorithm, whereas the optimal values of the design variables were determined by applying the Improved Minimum Distance Selection Method (IMDSM). Optimized designs were obtained for different values of the stiffness constraint between 0 and 500 N/mm. It was found that independently of the stiffness constraint value, the optimal value of the cell wall length ratio was 0.2 and the optimal cell wall thickness was 1.4 mm. These values correspond to the minimum cell wall length ratio and maximum cell wall thickness considered in this study, respectively. On the other hand, if the stiffness of the structure needs to be increased, both the cell wall angle and skin thickness must be increased accordingly. Furthermore, an increase in flexural stiffness is accompanied by an increase in both the mass and maximum load capacity of the structure.

## Figures and Tables

**Table 1:** Design parameters selected for sample manufacturing and testing.

#	Specimen ID	*h/l*	*θ* (°)	*t* (mm)	*t_f_* (mm)	Rel. Density	*c* (mm)	*m* (g)
1	S03300808	0.3	30	0.8	0.8	0.256	16.6	7.8
2	S03300820	0.3	30	0.8	2.0	0.256	16.6	11.0
3	S03301208	0.3	30	1.2	0.8	0.383	16.6	10.7
4	S03301220	0.3	30	1.2	2.0	0.383	16.6	13.8
5	S03400808	0.3	40	0.8	0.8	0.217	22.2	8.5
6	S03400820	0.3	40	0.8	2.0	0.217	22.2	11.8
7	S03401208	0.3	40	1.2	0.8	0.325	22.2	11.8
8	S03401220	0.3	40	1.2	2.0	0.325	22.2	14.9
9	S05300808	0.5	30	0.8	0.8	0.222	20.8	8.3
10	S05300820	0.5	30	0.8	2.0	0.222	20.8	11.5
11	S05301208	0.5	30	1.2	0.8	0.333	20.8	11.4
12	S05301220	0.5	30	1.2	2.0	0.333	20.8	14.7
13	S05400808	0.5	40	0.8	0.8	0.194	26.9	9.1
14	S05400820	0.5	40	0.8	2.0	0.194	26.9	12.3
15	S05401208	0.5	40	1.2	0.8	0.292	26.9	12.4
16	S05401208	0.5	40	1.2	2.0	0.292	26.9	15.8

**Table 2:** The 3D printed PLA mechanical properties, with standard deviation values in parentheses.

Material Property	Density (kg/m^3^)	Elastic Modulus (Gpa)	Yield Strength (Mpa)	Stress Plateau (Mpa)	Failure Strain
PLA	1240	2.2 (0.026)	34.9 (0.77)	26 (0.58)	0.52 (0.06)

**Table 3:** Summary of average mechanical properties (with standard deviations in parentheses) from three-point bending tests. The deformation energy refers to a vertical displacement of 5 mm.

#	Specimen ID	*P*_max_ [N]	*K* [N/mm]	*E* [N-mm]	*m* [g]
1	S03300808	172.9 (1.8)	124.9 (1.4)	610.7 (4.7)	8.27 (0.04)
2	S03300820	258.0 (4.3)	201.2 (3.6)	831.8 (14.9)	11.63 (0.03)
3	S03301208	355.0 (6.3)	241.1 (5.0)	1351.9 (29.3)	11.25 (0.02)
4	S03301220	529.6 (5.4)	359.3 (21.7)	1701.8 (3.1)	14.74 (0.08)
5	S03400808	180.0 (6.5)	141.0 (4.9)	648.0 (46.4)	9.04 (0.02)
6	S03400820	304.1 (13.5)	228.5 (7.8)	1140.8 (88.0)	12.47 (0.13)
7	S03401208	419.8 (8.0)	295.2 (13.6)	1595.7 (40.6)	12.78 (0.12)
8	S03401220	624.2 (0.9)	434.0 (8.8)	2336.2 (34.8)	16.24 (0.03)
9	S05300808	160.5 (4.0)	117.5 (5.7)	583.1 (7.9)	8.98 (0.03)
10	S05300820	232.6 (4.3)	166.5 (3.3)	912.1 (22.9)	12.31(0.15)
11	S05301208	343.0 (4.9)	234.5 (11.3)	1235.2 (21.5)	12.08 (0.07)
12	S05301220	482.5 (21.3)	339.5 (3.1)	1705.8 (51.3)	15.37 (0.12)
13	S05400808	158.5 (1.6)	123.6 (5.6)	605.5 (3.9)	9.83 (0.06)
14	S05400820	257.2 (7.2)	186.4 (7.4)	1027.8 (48.7)	13.14 (0.01)
15	S05401208	405.9 (4.5)	275.0 (6.0)	1498.5 (11.5)	13.6 (0.12)
16	S05401208	537.7 (3.0)	375.8 (4.9)	2097.6 (32.1)	16.89 (0.15)

**Table 4:** Summary of FE properties: maximum load, stiffness, deformation energy up to a 5 mm displacement, and mass (prediction error against average experimental results).

#	Specimen ID	*P*_max_ (N)	*K* (N/mm)	*E* (N-mm)	*m* (g)
1	S03300808	179.7 (3.9)	126.7 (1.4)	618.9 (1.3)	7.85 (5.1)
2	S03300820	284.9 (10.4)	216.3 (7.5)	912.0 (9.6)	11.01 (5.4)
3	S03301208	345.3 (2.7)	240.8 (0.1)	944.8 (30.1)	10.70 (4.9)
4	S03301220	491.0 (7.3)	386.6 (7.6)	1418.3 (16.7)	13.86 (5.9)
5	S03400808	205.8 (14.3)	162.3 (15.1)	720.4 (11.2)	8.56 (5.3)
6	S03400820	339.9 (11.8)	258.6 (13.2)	1085.7 (4.8)	11.82 (5.2)
7	S03401208	436.6 (4.0)	319.9 (8.4)	1292.8 (19.0)	11.82 (7.5)
8	S03401220	591.1 (5.3)	473.5 (9,1)	1707.8 (26.9)	14.98 (7.8)
9	S05300808	158.9 (1.0)	108.9 (7.3)	472.4 (19.0)	8.36 (6.9)
10	S05300820	267.5 (15.0)	172.1 (3.4)	1163.9 (27.6)	11.52 (6.4)
11	S05301208	328.0 (4.4)	243.3 (3.7)	980.9 (20.6)	11.42 (5.5)
12	S05301220	487.5 (1.0)	356.7 (5.1)	2344.5 (37.4)	14.68 (4.5)
13	S05400808	169.9 (7.2)	129.6 (4.5)	530.3 (12.4)	9.17 (6.7)
14	S05400820	304.5 (18.4)	193.3 (3.7)	1515.3 (47.4)	12.33 (6.2)
15	S05401208	398.1 (1.9)	297.3 (8.1)	1315.9 (12.2)	12.64 (6.9)
16	S05401208	584.3 (8.7)	413.8 (10.1)	2942.0 (40.3)	15.80 (6.5)

**Table 5:** Central and axial geometry specimens.

#	Specimen ID	*h/l*	*θ* (°)	*t* (mm)	*t_f_* (mm)	Rel. Density	*c* (mm)
1	S02351014	0.2	35	1.0	1.4	0.316	17.0
2	S04251014	0.4	25	1.0	1.4	0.324	16.3
3	S04350614	0.4	35	0.6	1.4	0.164	21.4
4	S04351014	0.4	35	1.0	1.4	0.274	21.4
5	S04351024	0.4	35	1.0	2.4	0.274	21.4
6	S04351414	0.4	35	1.4	1.4	0.383	21.4
7	S04451014	0.4	45	1.0	1.4	0.241	28.2
8	S06351014	0.6	35	1.0	1.4	0.246	25.8

**Table 6:** Results of the regression analysis.

Regression Model	Coefficients	*R* ^2^	*RMS*
Mass (g)	*A*_1_ = 2.802, *A*_2_ = 5.672, *A*_3_ = 2.904	0.988	0.246
Stiffness (N/mm)	*C*_1_ = 1.433, *C*_2_ = 5.202, *C*_3_ = 3.100, *C*_4_ = 0.172, *C*_5_ = 6.361	0.942	23.9
Maximum Load (N)	*D*_1_ = −164.960, *D*_2_ = 240.510, *D*_3_ = 35.113, *D*_4_ = 1.097, *D*_5_ = 2.014	0.962	29.6

**Table 7:** Pareto optimal design variables and corresponding properties (FE validation).

Stiffness Constraint	*h/l*	*θ* (°)	*t* (mm)	*t_f_* (mm)	Mass (g)	*P*_max_ (N)	*K* (N/mm)
*K*_min_ = 0	0.2	25.1	1.4	0.8	11.91	514.8 (380.8)	390.3 (221.0)
*K*_min_ = 100 (N/mm)	0.2	30.7	1.4	0.8	12,43	542.6 (431.0)	389.5 (272.7)
*K*_min_ = 200 (N/mm)	0.2	40.9	1.4	0.8	13.82	617.8 (542.0)	378.1 (385.5)
*K*_min_ = 300 (N/mm)	0.2	44.9	1.4	0.8	14.61	660.5 (606.8)	370.3 (451.9)
*K*_min_ = 400 (N/mm)	0.2	45.0	1.4	1.0	15.36	678.3 (623.8)	400.2 (490.6)
*K*_min_ = 500 (N/mm)	0.2	37.4	1.4	2.4	17.75	770.4 (786.2)	577.6 (632.3)

## Data Availability

The original contributions presented in the study are included in the article.
